# Screening of potential vaccine candidates against pathogenic *Brucella* spp. using compositive reverse vaccinology

**DOI:** 10.1186/s13567-021-00939-5

**Published:** 2021-06-02

**Authors:** Xiaodong Zai, Ying Yin, Fengyu Guo, Qiaoling Yang, Ruihua Li, Yaohui Li, Jun Zhang, Junjie Xu, Wei Chen

**Affiliations:** Laboratory of Vaccine and Antibody Engineering, Beijing Institute of Biotechnology, Beijing, China

**Keywords:** *Brucella* spp., Reverse vaccinology, Core proteome, Broad-spectrum antigen, Vaccine candidate

## Abstract

*Brucella* spp. are Gram-negative, facultative intracellular bacteria that cause brucellosis in humans and various animals. The threat of brucellosis has increased, yet currently available live attenuated vaccines still have drawbacks. Therefore, subunit vaccines, produced using protein antigens and having the advantage of being safe, cost-effective and efficacious, are urgently needed. In this study, we used core proteome analysis and a compositive RV methodology to screen potential broad-spectrum antigens against 213 pathogenic strains of *Brucella* spp. with worldwide geographic distribution. Candidate proteins were scored according to six biological features: subcellular localization, antigen similarity, antigenicity, mature epitope density, virulence, and adhesion probability. In the RV analysis, a total 32 candidate antigens were picked out. Of these, three proteins were selected for assessment of immunogenicity and preliminary protection in a mouse model: outer membrane protein Omp19 (used as a positive control), type IV secretion system (T4SS) protein VirB8, and type I secretion system (T1SS) protein HlyD. These three antigens with a high degree of conservation could induce specific humoral and cellular immune responses. Omp19, VirB8 and HlyD could substantially reduce the organ bacterial load of *B. abortus* S19 in mice and provide varying degrees of protection. In this study, we demonstrated the effectiveness of this unique strategy for the screening of potential broad-spectrum antigens against *Brucella*. Further evaluation is needed to identify the levels of protection conferred by the vaccine antigens against wild-type pathogenic *Brucella* species challenge.

**Supplementary Information:**

The online version contains supplementary material available at 10.1186/s13567-021-00939-5.

## Introduction

*Brucella* spp. are Gram-negative, facultative intracellular bacteria that cause brucellosis in humans and various animals [1]. The genus *Brucella* comprises a growing number of species (at least 12) that infect a wide variety of mammals as primary hosts [2, 3]. Brucellosis is one of the most common zoonotic diseases worldwide and has become a serious concern in recent years [4]. At present, vaccination is the most effective approach to preventing and controlling brucellosis. Veterinary live attenuated vaccines have been widely used and play an important role in the control of brucellosis epidemics [5]. However, this bacterium remains a challenging vaccine target that due to some drawbacks shown by these live attenuated vaccines, including interference with diagnostic tests, pathogenicity for humans, potential to cause abortion in pregnant animals, among others [6]. Subunit vaccines have promising applications with the advantage of being safe, cost-effective and efficacious. During the past two decades, various antigens have been extracted from *Brucella*, such as Omp19, Omp25, L7/L12, P39, SodC, InpB, AsnC and TF [7–16]. These available antigens have been shown to provide protection against *Brucella* infection by reducing the organ bacterial load in mice. Whereas such findings are highly promising, subunit vaccines using known antigens cannot provide the levels of protection conferred by live attenuated vaccines [17]. Further investigation is needed to identify novel antigens, so as to increase vaccine efficacy. *B. abortus*, *B. melitensis*, and *B. suis* are considered the most highly pathogenic species, causing most cases of brucellosis in humans and domestic animals throughout Central Asia, Africa, South America, and the Mediterranean region [4]. It is of great importance to select broad-spectrum antigens that can simultaneously target various *Brucella* pathogens with a worldwide geographic distribution.

Reverse vaccinology (RV) has been proven to be a highly effective approach in which a rational vaccine design is used, with vaccine antigen prediction based on bioinformatics analysis of pathogen genomes [18, 19]. Several studies have used RV to screen potential vaccine candidates based on the protein coding genome of *Brucella* [20–23]. Different selection criteria with traditional rules-based prediction have been applied, resulting in the identification of numerous potential antigens. However, the “all-or-nothing” type of selection used in these studies might fail to capture the relationship among different criteria because each rule is important but not decisive [24, 25]. Moreover, these early studies typically analysed few representative strains that are unfavourable targets for broad-spectrum therapeutics.

The objective of the present study was to screen potential broad-spectrum antigens against a large sample of globally representative strains of pathogenic *Brucella* spp. using a compositive RV strategy. To address this objective, we adopted an in silico methodology for selecting novel potential vaccine candidates based on their biological features that are strongly associated with protective antigenicity. From these in silico predictions, 32 potential vaccine candidates from the core proteome of *Brucella* were picked out. Outer membrane protein Omp19, type IV secretion system (T4SS) protein VirB8, and type I secretion system (T1SS) protein HlyD were then selected for preliminary verification in a mouse model. Our results provided a manageable list of potential protective antigens for developing a broad-spectrum vaccine against *Brucella* spp. with a worldwide geographic distribution.

## Materials and methods

### Bioinformation and reverse vaccinology

The genome of *B. abortus*, *B. melitensis*, and *B. suis* strains with clear geographic characteristics were selected and downloaded from the NCBI website (as of March 2019) [26]. To identify the pan-core proteome, we used an ultra-fast computational pipeline Bacterial Pan Genome Analysis (BPGA) tool with default parameters [27]. The protein FASTA files of all strains were input for orthologous cluster analysis, with an 80% sequence identity cut-off value. The core/accessory/unique proteomes were defined as coverage > 95%, 95–15%, and  < 15%, respectively.

Proteins from the core proteome were first aligned with host (human/mouse) protein databases using the BLASTp tool [28]. Then, non-host-homology proteins were screened and scored according to a compositive strategy assigning six biological features: (1) subcellular localization (SCL), (2) antigen similarity, (3) antigenicity, (4) mature epitope density, (5) virulence, and (6) adhesion probability. Each of the six biological features was used to divide proteins into three levels of antigen probability: high probability (individual score = 1), moderate probability (individual score = 0.5), and low probability (individual score = 0). Then, all proteins were computed and ranked based on a predicted composite score of the six individual scores for further analysis. The remaining top-ranked (1% of the core proteome) proteins were considered potential vaccine candidates.

Briefly, the CELLO program was used to predict the most likely location for each protein and a subcellular localization individual score was obtained (extracellular/membrane, score = 1; periplasmic, score = 0.5; cytoplasmic, score = 0) [29]. For computation of antigen similarity, we made use of sequence similarity search programs in BLAST to identify similar sequences in the target of known protective antigens database (Protegen, exclude *Brucella* antigens) and obtained a similarity individual score (similarity ≥ 200, score = 1; 100 ≤ similarity < 200, score = 0.5; similarity < 100, score = 0) [30, 31]. For antigenicity computation, we used an alignment-free approach (VaxiJen) to antigen prediction based on principal amino acid properties and obtained an antigenicity individual score (antigenicity ≥ 0.6, score = 1; 0.45 ≤ similarity < 0.6, score = 0.5; similarity < 0.45, score = 0) [32]. A computational strategy (MED, mature epitope density) based on measuring the epitope concentration in the mature protein according to the number of 9-mer epitopes was used to obtain a MED individual score (density ≥ 10, score = 1; 2 ≤ density < 10, score = 0.5; density < 2, score = 0) [33]. The virulence probability of proteins was predicted with MP3 using an integrated support vector machine and hidden Markov model approach to obtain a virulence individual score (virulence ≥ 0.5, score = 1; 0 ≤ virulence < 0.5, score = 0.5; virulence < 0, score = 0) [34]. Adhesion probability of the input proteins was predicted using the Vaxign tool and SPAAN program that obtained an adhesion individual score (adhesion ≥ 0.4, score = 1; 0.3 ≤ density < 0.4, score = 0.5; density < 0.3, score = 0) [35].

### Protein structure and function

To gain greater insight into the biological functions of these candidate antigens, proteins were manually annotated using eggNOG-mapper [36]. The protein structure of the antigens Omp19, VirB8, and HlyD was generated with Phyre2 tools using a homology modelling approach [37]. A comparative analysis of candidate antigens in this study and previous studies was conducted and visualized using Circoletto [38]. For the convenience of researchers, we mostly used publicly available web servers for bioinformatics and immune-informatics analyses in this study.

### Bacterial strains

The *B. abortus* strains 104 M and S19 were preserved in our laboratory (Beijing Institute of Biotechnology, Beijing, China). *B. abortus* 104 M has been widely used in China for the control and prevention of human brucellosis since its approval by the Chinese Food and Drug Administration (CFDA) in 1965. It exhibits typical properties of biotype I, low and stable virulence in experimental animals, and strong immunogenicity [39, 40]. *B. abortus* strain S19 (also called A19 in China), which can infect humans, causing typical features of brucellosis, was originally isolated as a virulent strain from a Jersey cow in 1923 [41]. *B. abortus* was cultured in Tryptic soy broth (TSB) with continuous shaking (220 rpm) at 37 °C. Strains of *Escherichia coli* Trans1-T1 and BL21 (DE3) (TransGen Biotech Co., Ltd., Beijing, China), used for cloning and expression studies, were grown in Luria–Bertani medium. All experiments involving live *B. abortus* strains were performed in approved and registered biosafety facilities as stipulated. We strictly followed standard biosafety procedures and protocols throughout the entire process of the experiments.

### Cloning and expression of antigens

Three potential protective antigens of *Brucella* were selected for evaluation: Omp19, VirB8, and HlyD. Gene coding for the proteins was cloned from *B. abortus* 104 M as C-terminal His-tagged fusion protein and then expressed and purified as previously reported, with some modifications [42]. Briefly, the PCR products of target genes (without signal peptides) were purified and then cloned into a pEASY-Blunt E1 expression vector (TransGen). *E. coli* BL21 (DE3) cells containing the expression vector were grown at 37 °C to an OD600 of ~0.8 and induced with isopropyl-β-D-thiogalactopyranoside (IPTG) at a final concentration of 1 mM at 25 °C for 6 h. The recombinant mature proteins were expressed as soluble proteins that were purified using HisTrap HP (GE Healthcare, Uppsala, Sweden) chromatography according to the manufacturer’s instructions. After purification, the purity of the proteins was analysed on 12% gels using SDS-PAGE (Bio-Rad, Hercules, CA, USA) and detected with anti-His tag antibody in Western blot analysis. The protein concentration was estimated using BCA assay (R&D System, Minneapolis, MN, USA) and stored at −80 °C for future assays.

### Antigen immunization

To investigate the immune protection effect of candidate antigens, a mouse model was used in this study, as previously reported [5, 43]. Six- to eight-week-old female C57BL/6 J mice were purchased from Charles River Laboratories, Inc (Beijing, China). and randomly divided into five treatment groups (*n* = 10). Mice were immunized subcutaneously with a prime and two boosts (0, 14, 28 days) of vaccine formulation containing 20 μg of protein (Omp19, VirB8, or HlyD) in phosphate-buffered saline (PBS), combined with complete Freund's adjuvant (Sigma-Aldrich, St. Louis, MO, USA) at day 0 and incomplete Freund’s adjuvant at day 14/28. The positive control group was intraperitoneally immunized with 1 × 10^5^ CFU (colony-forming unit) of live attenuated *B. abortus* vaccine strain 104 M once at day 0, and the negative control group was injected with sterile 1X PBS (pH 7.4). Blood was collected via retro-orbital bleeding on days 0, 14, 28, and 35 of the immunization schedule.

### Detection of antibodies and cytokines

To determine the titre of antibody (IgG, IgG1 and IgG2a) specific to antigens in the serum of immunized mice, a standard enzyme-linked immunosorbent assay (ELISA) was used as explained before with some changes [8]. Briefly, 96-well polystyrene plates (Corning; New York, NY, USA) were coated with purified recombinant proteins (100 μL, 2 μg/mL) at 4 °C overnight. After incubation, plates were washed four times with PBS containing 0.1% Tween-20 (PBST) and blocked with PBST containing 2% bovine serum albumin (Sigma-Aldrich) for 1 h at 37 °C. Plates were then incubated with appropriate dilutions of the different samples for 1 h at 37 °C. After washing, as above, plates were incubated with secondary horseradish peroxidase-conjugated anti-mouse total IgG or different IgG subclasses (IgG1 and IgG2a) (Cell Signaling Technology, Danvers, MA, USA) for 1 h at 37 °C. Plates were then washed and 100 μL 3,3′,5,5′-tetramethylbenzidine dihydrochloride (TMB) substrate solution (Solarbio, Beijing, China) was added. After 7 min of incubation at room temperature, the reaction was stopped with 1 M H_2_SO_4_. The OD at 450 nm/630 nm was read on a microplate reader (BioTek; Winooski, VT, USA).

Levels of cytokine tumour necrosis factor α (TNF-α), interferon-γ (IFN-γ), interleukin-4 (IL-4), IL-6, and IL-10, and IL-17 in the supernatants from stimulated splenocytes were detected using a cytometric bead array (CBA Mouse Cytokine Kit; BD Biosciences, San Jose, CA, USA), according to the manufacturer’s instructions. Briefly, spleens from antigen-immunized mice were collected 4 weeks after the third immunization and homogenized in sterile PBS. After removal of red blood cells, splenocytes at a concentration of 2 × 10^6^/mL were added to a 96-well microplate with RPMI 1640 medium containing 5 µg/mL antigen protein and supplemented with 10% foetal calf serum (HyClone Laboratories, Logan, UT, USA). Concanavalin A at 5 µg/mL was used as a positive control. The supernatant was collected to evaluate cytokines after stimulation for 18 h.

### Protection experiments

One week after the third immunization, mice from each group (*n* = 6) were challenged intraperitoneally with *B. abortus* strain S19 (1 × 10^6^ CFU) [43]. Two weeks post challenge, the spleens and livers of infected mice were removed and homogenized. The bacterial load in organ tissues was determined following serial dilution of homogenates in sterile 1X PBS, plating on blood agar, and incubation for 3 days at 37 °C. The CFUs in spleens and livers were determined and converted to logarithmic values. The unit of protection conferred by antigen proteins was obtained by subtracting the mean log_10_CFU of the experimental group from that of the corresponding PBS group.

### Statistical analyses

In the immunogenicity study, antibody titres, cytokine expression and Protection units were analysed using standard t-tests and one-way ANOVA performed using GraphPad Prism 7.0 (GraphPad Software, San Diego, CA, USA). A *p* value < 0.05 was considered significant. Two independent experiments were conducted in this study. The data are expressed as mean ± standard deviation of multiple mice in single group from one independent experiment.

## Results

### Pan-core proteome of pathogenic *Brucella* spp. with worldwide geographic distribution

**Figure 1:**
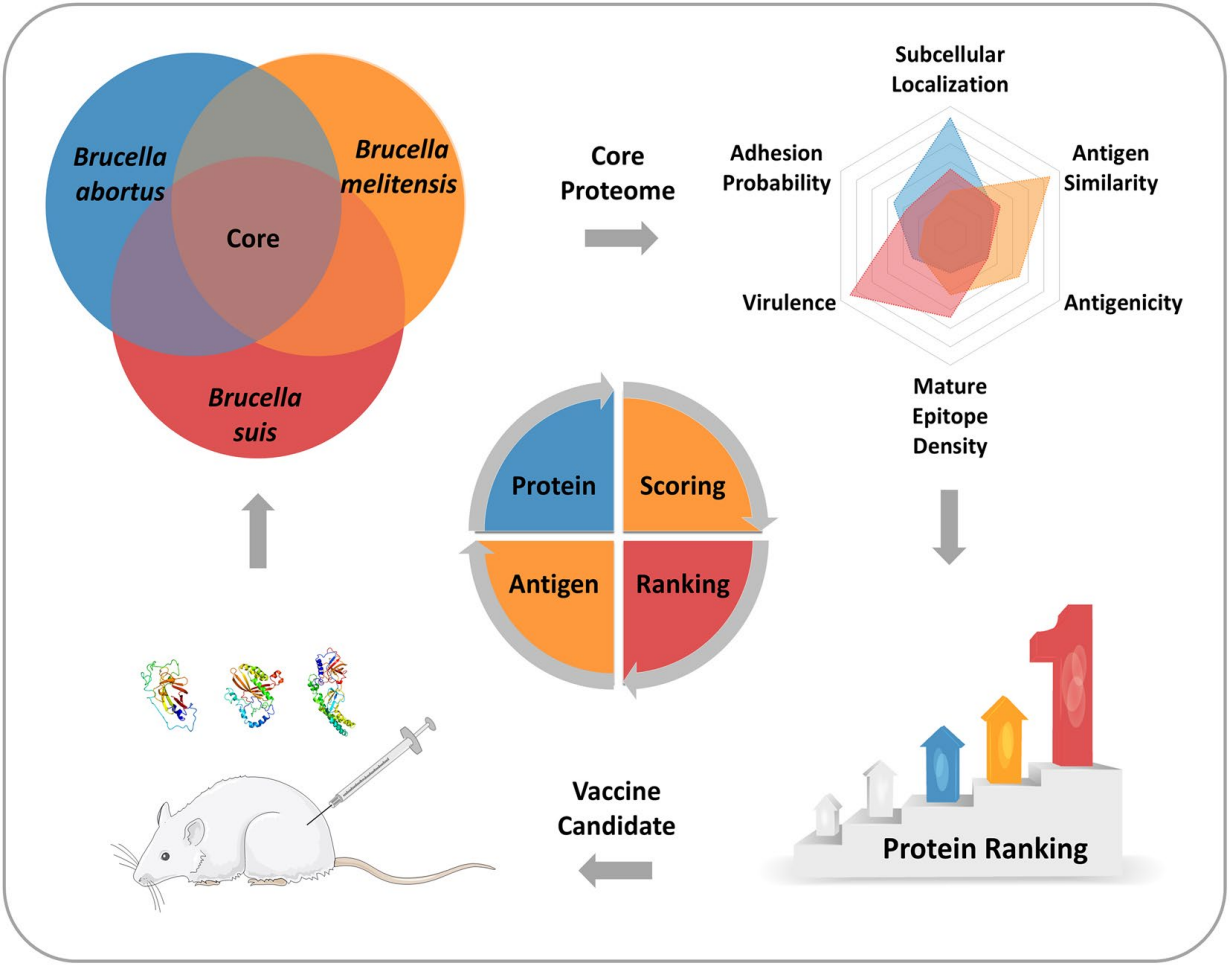
**Schematic representation of reverse vaccinology (RV) approaches applied in this study**. Pathogenic *B. abortus*, *B. melitensis*, and *B. suis* strains with worldwide geographic distribution were analysed using an RV strategy. Non-host-homology proteins from the core proteome were scored and ranked based on their predicted composite score, and top-ranked proteins were selected as potential vaccine candidates. Three vaccine candidates were evaluated for immunogenicity and protective efficacy in mice.

**Figure 2:**
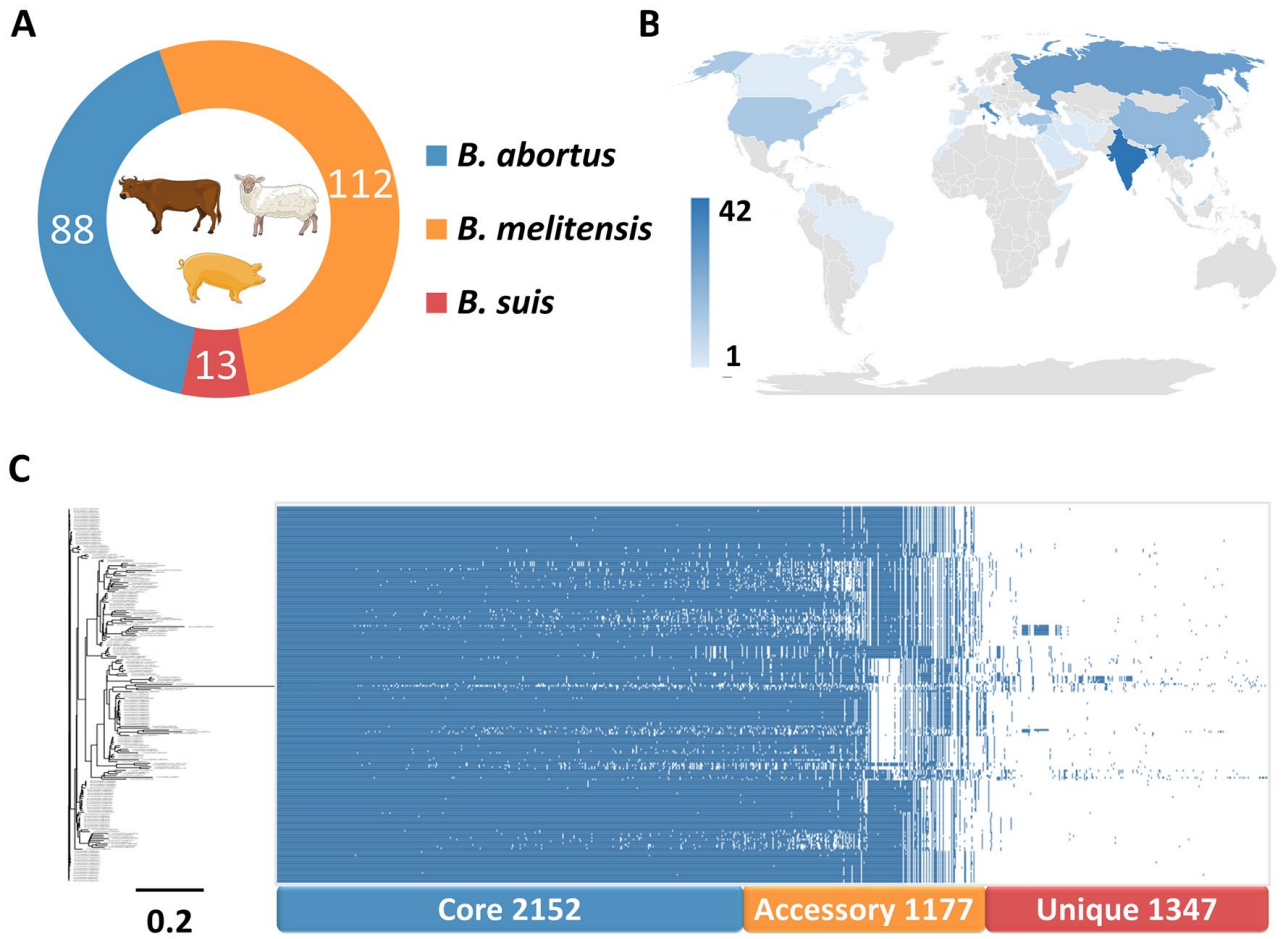
**Pan-core proteome analysis of pathogenic* Brucella* spp. with worldwide geographic distribution**. **A** A total 213 strains of *B. abortus*, *B. melitensis*, and *B. suis* with clear genetic isolation information were selected in pan-core proteome analysis. **B** Representative strains covering major brucellosis-epidemic areas such as Asia, Europe and the Americas. **C** A genome-based neighbour-joining phylogenetic tree was constructed based on concatenated core protein alignments. The core/accessory/unique proteome was defined as coverage > 95%, 95–15%, and < 15%, respectively.

To screen potential broad-spectrum vaccine candidates against pathogenic *Brucella* spp. (*B. abortus*, *B. melitensis*, and *B. suis*) that are geographically distributed worldwide, core proteome analysis followed by a compositive RV strategy was used in this study (Figure 1). To identify the pan-core proteome of pathogenic *Brucella* spp. with global geographic distribution, 213 strains of *B. abortus*, *B. melitensis*, and *B. suis* with clear genetic isolation information were selected after retrieval from NCBI (Figure 2A, Additional file 1). These strains have extensive representation that cover 20 countries distributed throughout Asia, Europe, the Americas, and other brucellosis-epidemic areas, highlighting challenges in the design of vaccines with global utility (Figure 2B). The protein presence/absence matrix resulted in a pan proteome with 4676 proteins and a core proteome with 2152 proteins, based on an 80% sequence identity threshold (Figure 2C).

### Identification of novel *Brucella* antigens using reverse vaccinology (RV) approaches

**Figure 3:**
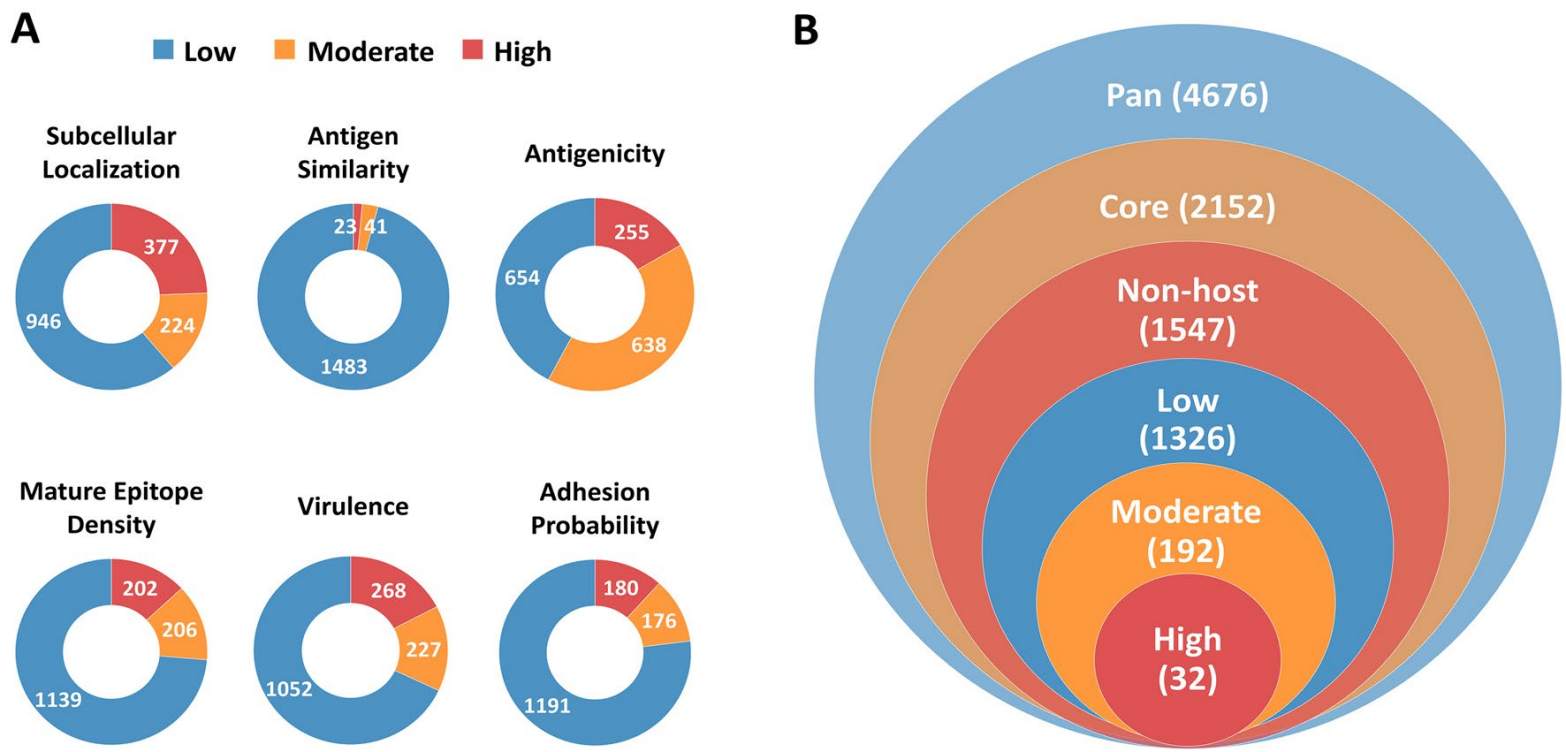
**Distribution of individual and composite scores for *Brucella* core proteome**. **A** Each of six biological features were used to divide proteins into three levels of antigen probability: high, moderate, and low probability. **B** All proteins were computed based on a composite score of the six individual scores. The top 32 proteins were selected as potential vaccine candidates, screened from the pan proteome of 4676 proteins.

Any proteins from the core proteome exhibiting sequence homology with host (human and mouse) proteins were removed. The residual non-host-homology proteins (1547) were then scored according to six biological features that are strongly associated with protective antigenicity, as follows (Figure 3A).

#### Subcellular localization (SCL)

Because the antibody responses induced by protective antigens are primarily located in the extracellular or membranes, the SCL of antigens is a main selection criterion in RV strategies [24, 29]. Here, a total of 377, 224, and 946 proteins were predicted to located in extracellular/membrane (high), periplasmic (moderate) and cytoplasmic (low), respectively.

#### Antigen similarity

One of the most common ways to identify new potential antigens is in a comparison of similarity [30, 44]. This approach was developed based on the assumption that some antigens are protective, in that they have specific structural/functional features (“protective signatures”) that distinguish them from immunologically irrelevant pathogen components. Here, a total of 23, 41, and 1483 proteins showed high, moderate, and low similarity to known antigens, respectively.

#### Antigenicity

Because alignment-dependent sequence similarity methods have limitations in the discovery of truly novel antigens, we used a novel alignment-independent method for antigen recognition based on the main chemical properties of amino acid sequences [32]. Here, a total of 255, 638, and 654 proteins showed high, moderate, and low antigenicity, respectively.

#### Mature epitope density

We also used a computational strategy to predict target proteins for RV according to the number of epitopes in the mature protein portion, considering the epitope’s concentration in this portion, called mature epitope density (MED) [33]. Here, a total of 202, 206, and 1139 proteins showed high, moderate, and low mature epitope density, respectively.

#### Virulence

Virulence factors are proteins that can aid colonize a host organism and/or induce disease. Using these antigenic factors as vaccine candidates might increase vaccine efficiency as these proteins are frontline weapons in the pathogenic arsenal [44]. Here, a total of 268, 227 and 1052 proteins had high, moderate, and low probability of being a virulence factor, respectively.

#### Adhesion probability

Adhesion is critical for bacterial invasion and the capacity to induce strong immune responses [45]. Adhesion of *Brucella* to extracellular matrix components or to host cells is an important step in infection [46]. Several adhesins have been identified in *Brucella* and shown to provide protection in immunization experiments [47]. Here, a total of 180, 176, and 1191 proteins has high, moderate, and low adhesion probability, respectively.

Next, all proteins were computed based on a composite score of the six individual scores (listed in Additional file 2). Subsequently, the 32 top-ranked proteins (1% of the core proteome, with a composite score ≥ 0.75) were selected for consideration as high-probability potential vaccine candidates, screened from the core proteome (Figure 3B).

As shown in Table 1, the potential antigens could be divided into three groups according to their composite scores (0.916, 0.833 and 0.75). Two antigens (Omp19 and Omp25) achieved a composite score of 0.916, both of which were already identified as vaccine antigens against *Brucella* in numerous studies. Nine antigens (InpB, Omp10, VirB8, TolC, and so on) achieved a composite score of 0.833. The remaining 21 antigens (GumB, FusB, YgaZ, VirB6, HlyD, and so on) achieved a composite score of 0.75. These 32 antigens tended to fall into a few categories of biological function, including outer membrane proteins and secretory system proteins.

**Table 1:** **Top 32 potential vaccine candidates of *Brucella * identified using reverse vaccinologyrucella**

Accession number	Protein	Annotation	Length	Composite scores
WP_002964998.1	Omp19	Outer membrane lipoprotein omp19	177	0.917
WP_002963844.1	Omp25	Outer membrane lipoprotein omp25	213	0.917
WP_002963504.1	InpB	Invasion associated locus B protein	173	0.833
WP_002964778.1	–	Surface antigen	274	0.833
WP_002966502.1	Omp10	Outer membrane lipoprotein omp10	126	0.833
WP_002966517.1	VirB8	Type IV secretion system protein virB8	239	0.833
WP_002966799.1	TolC	Outer membrane efflux protein tolC	456	0.833
WP_002971524.1	LppA	Surface antigen	150	0.833
WP_002964333.1	YiaD	Cell envelope biogenesis protein	220	0.833
WP_002965367.1	–	Outer membrane autotransporter barrel domain-containing protein	230	0.833
WP_002965368.1	–	Outer membrane autotransporter barrel domain-containing protein	228	0.833
WP_002963919.1	GumB	Sugar ABC transporter substrate-binding protein	195	0.75
WP_002964886.1	FusB	Fusaric acid resistance protein FusB	176	0.75
WP_002964930.1	YgaZ	Branched-chain amino acid ABC transporter permease	224	0.75
WP_002967278.1	TcyB	Cysteine ABC transporter permease	226	0.75
WP_002969266.1	VirB6	Type IV secretion system protein VirB6	347	0.75
WP_002969595.1	AsmA	Cell envelope biogenesis protein AsmA	645	0.75
WP_002964765.1	MacA	Membrane fusion protein	408	0.75
WP_002971258.1	HlyD	Hemolysin secretion protein D	349	0.75
WP_002966947.1	Omp16	Outer membrane lipoprotein omp16	168	0.75
WP_002966591.1	BhuA	Heme transporter BhuA	661	0.75
WP_002966636.1	FliC	Flagellin	282	0.75
WP_002969562.1	–	Uncharacterized protein	155	0.75
WP_002963954.1	–	Membrane protein	89	0.75
WP_002965055.1	–	Uncharacterized protein	339	0.75
WP_002963439.1	–	Hemolysin secretion protein	383	0.75
WP_002963645.1	–	Lipoprotein	192	0.75
WP_002963994.1	–	Aspartic protease	234	0.75
WP_002964835.1	–	Uncharacterized protein	153	0.75
WP_002964883.1	–	Lipoprotein	193	0.75
WP_002970401.1	–	Uncharacterized protein	339	0.75

### Comparative analysis of potential vaccine candidates with previous studies

**Figure 4:**
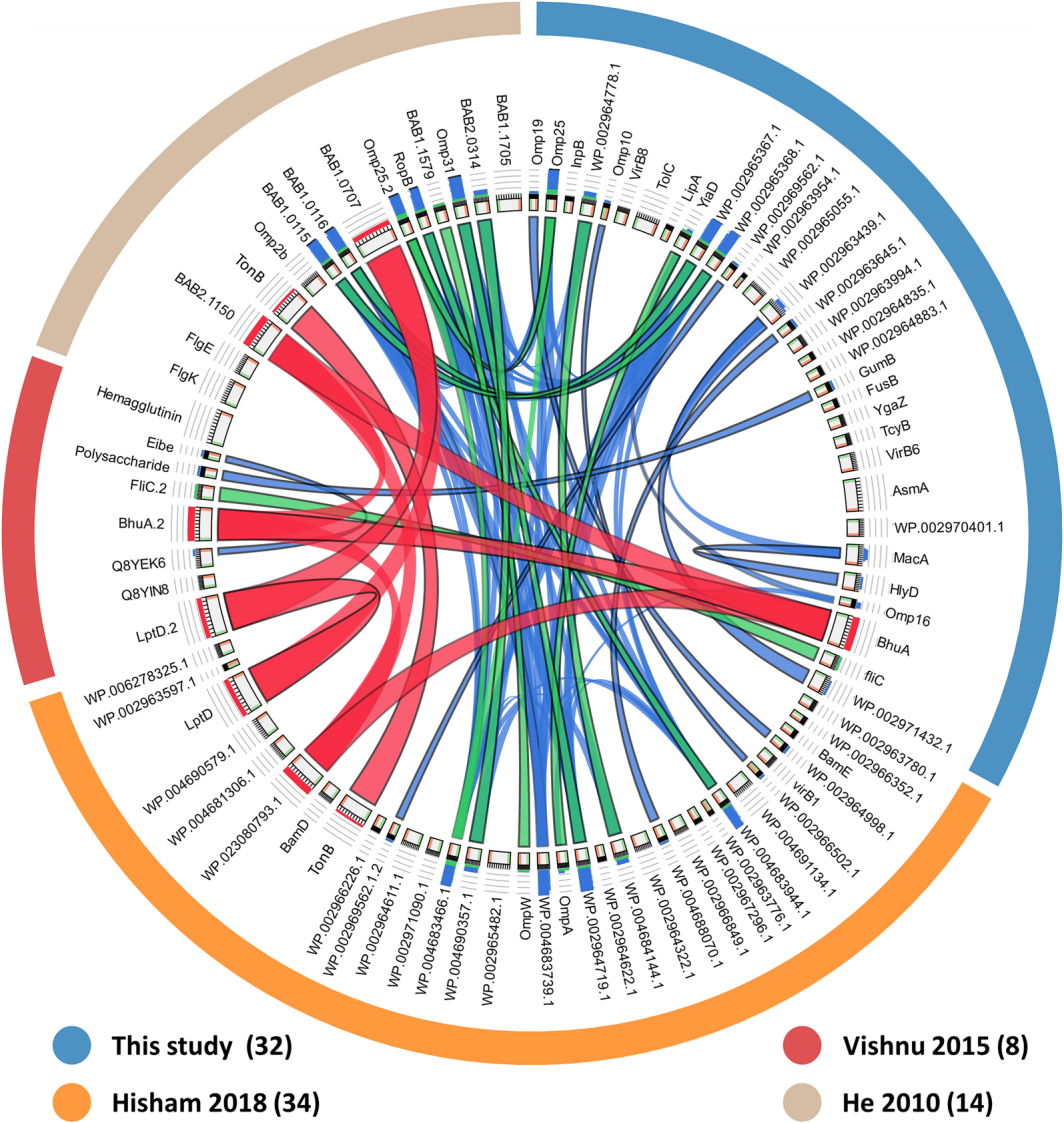
**Comparative analysis of potential vaccine candidates with previous studies**. Protein sequences of candidate antigens in this study and previous studies were translated into an all-against-all BLAST run for sequence comparison. Inside the circle, ribbons represent local alignments. BLAST results were produced using three colours (blue, green and red), representing quartiles, up to the maximum score.

Different selection criteria have been applied to *Brucella* vaccine antigen prediction, resulting in the selection of numerous potential antigens. By contrasting the similarity and dissimilarity of the candidate antigens identified in various studies, we observed similarities among the different RV strategies (Figure 4). Hisham and Ashhab obtained 34 potential antigens against pathogenic *Brucella* spp. according to an RV strategy using the cumulative score of surface-associated proteins [23]. Vishnu et al. identified potential vaccine candidates against *B. melitensis* 16 M based on systematic screening of the exoproteome and secretome, in which eight proteins were identified as potential vaccine candidates [22]. He and Xiang used the web-based VIOLIN vaccine target prediction program Vaxign to predict new *Brucella* vaccine targets, which identified 14 outer membrane proteins [20]. Many antigens reported by Hisham and Ashhab [23], Vishnu et al. [22] and He and Xiang [20] were also identified in our list, such as Omp25 and BhuA. While several non-broad-spectrum antigens identified in previous studies were absent from our antigen list, such as TonB-dependent receptor. Our results also provided a manageable list that includes some novel potential antigens that have not been previously reported, such as outer membrane efflux protein TolC, T1SS protein HlyD, T4SS protein VirB6/VirB8, aromatic acid exporter family protein FusB, and cell envelope biogenesis protein AsmA.

### Construction, expression, and purification of antigen proteins

**Figure 5:**
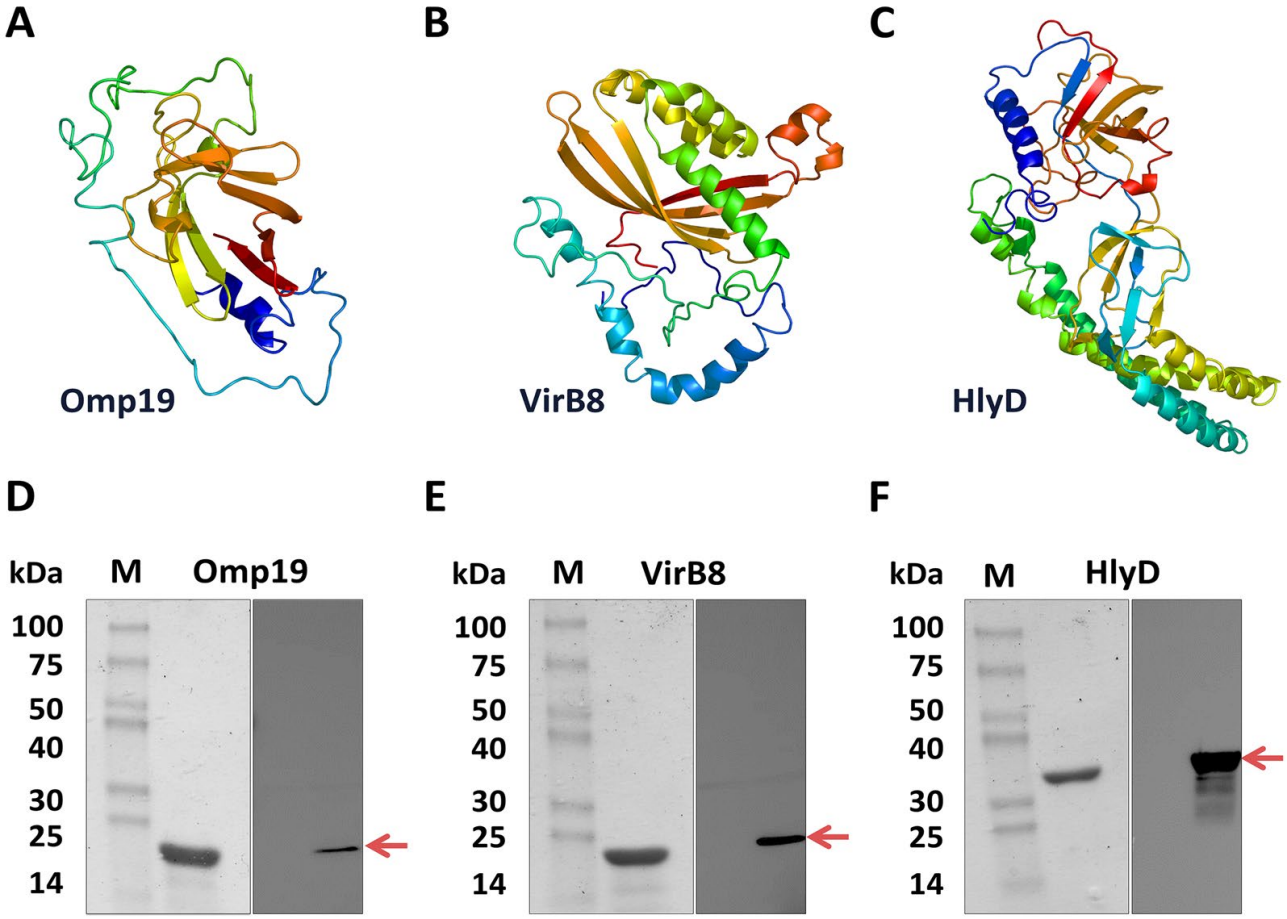
**Protein structure and expression of *Brucella* antigens Omp19, VirB8, and HlyD**. **A**–**C** The protein structure of the antigens Omp19, VirB8, and HlyD was generated with Phyre2 tools using a homology modelling approach. **D–F** SDS-PAGE and Western blot analysis of purified mature proteins. Omp19, VirB8, and HlyD were cloned as C-terminal His-tagged fusion proteins and then expressed and purified.

To demonstrate the effectiveness of the present RV methodology, three potential protective antigens with representative composite scores and families, including Omp19 (score 0.917, used as a positive control), VirB8 (score 0.833), and HlyD (score 0.75), were selected for preliminary verification. Omp19 is a surface-exposed lipoprotein broadly expressed within the *Brucella* genus, which plays an active role in bacterial cell adhesion, cell invasion, colonization, immune modulation, and intracellular survival (Figure 5A) [48]. It has demonstrated that Omp19 is a possible vaccine candidate and has adjuvant activity as protease inhibitor, which can be used as positive control [49, 50]. VirB8 from *Brucella* is a bitopic inner membrane protein, which undergoes several protein–protein interactions that have an impact on both the functionality and assembly of the T4SS complex, suggesting a key role in the T4S system (Figure 5B) [51, 52]. HlyD is a component of the prototypical alpha-hemolysin (HlyA) bacterial type I secretion T1S system, along with the other components HlyB and TolC (Figure 5C) [53]. Multiple-sequence alignment of Omp19, VirB8, and HlyD from the 213 pathogenic strains of *Brucella* spp. showed a low naturally occurring sequence variation and high (> 95%) coverage across this diverse population, suggesting strong evolutionary pressure, which in turn suggests the biological importance of target proteins. The mature proteins of interest were expressed and purified in high yields as C-terminal His-tag fusion proteins. The molecular weights were the correct size for Omp19 (17.8 kDa), VirB8 (21.2 kDa), and HlyD (36.3 kDa), respectively, as evidenced in SDS-PAGE and Western blot assays (Figures 5D–5F).

### Immunization of mice with novel antigens provides protection against *B. abortus* infection

**Figure 6:**
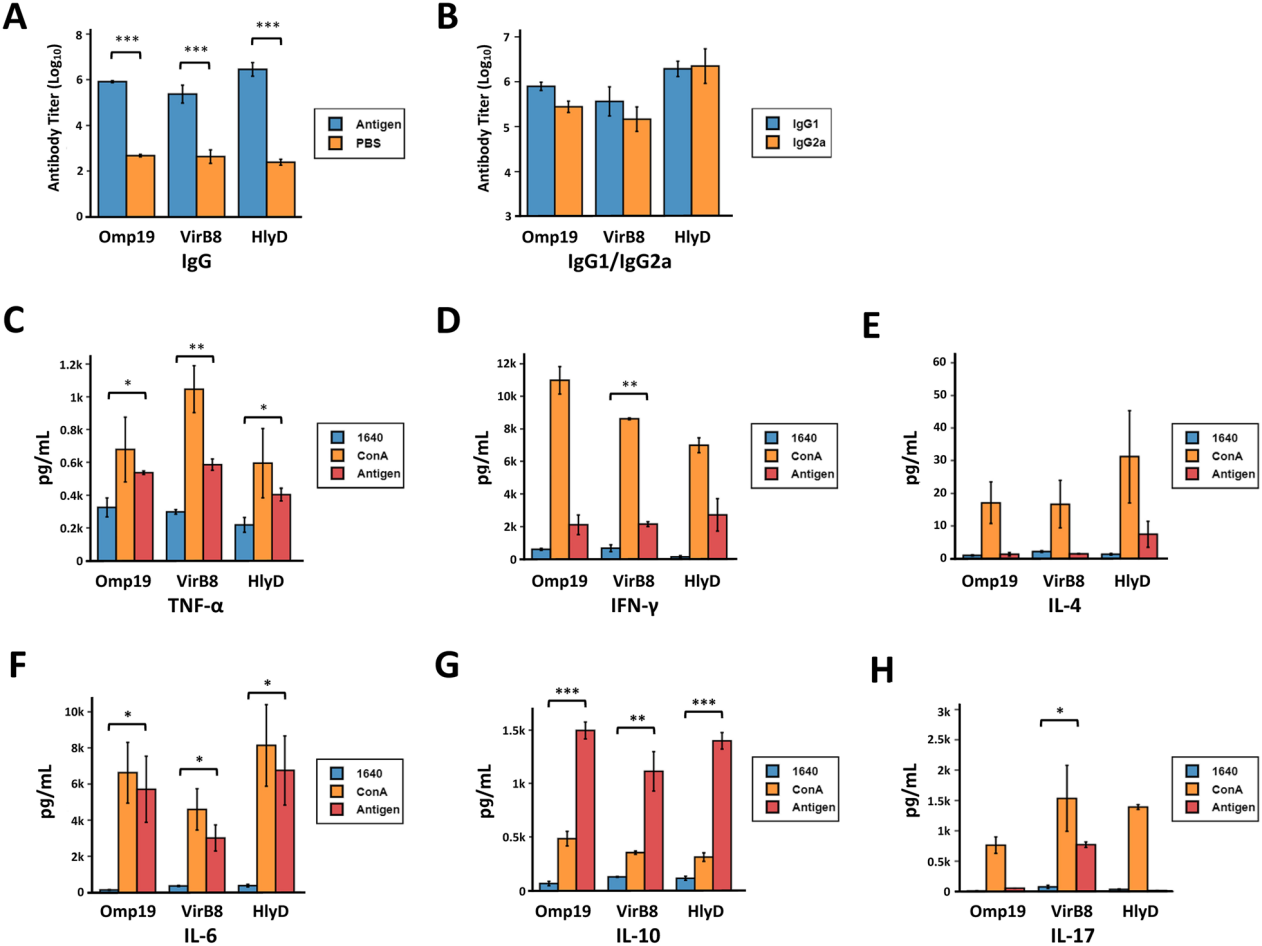
**Immunogenicity of candidate protective antigens Omp19, VirB8, and HlyD**. **A**, **B** Anti-antigen IgG titres and antibody subclasses in serum were determined by ELISA and represented as dilution titres. **C**–**H** Cellular immune responses elicited by antigen proteins. Expression levels of TNF-α, IFN-γ, IL-4, IL-6, IL-10 and IL-17 in supernatants were subsequently quantified. Error bar, mean ± S.D.

To investigate the immune protection effect of the candidate antigens Omp19, VirB8, and HlyD, a C57BL/6 J mouse model was used in this study. The specific antibody levels in serum and cytokines in the culture supernatant of mouse spleen cells were measured to evaluate the immunogenicity of the candidate protective antigens. Omp19, VirB8, and HlyD antigen proteins were observed to induce total IgG antibody titres of 8.2 × 10^5^, 3.0 × 10^5^, and 3.3 × 10^6^ after the third immunization, respectively (Figure 6A). At the same time, all three antigens could effectively elevate IgG1 and IgG2a levels (Figure 6B).

In brucellosis, different inflammatory cytokines work together to regulate the host immune system [54]. The level of cytokines TNF-α, IFN-γ, IL-4, IL-6, IL-10 and IL-17 in the supernatants of stimulated splenocytes were detected via Omp19, VirB8, and HlyD antigen protein stimulation (Figures 6C–H). Splenocytes from Omp19-, HlyD-, and VirB8-immunized mice secreted significantly higher amounts of TNF-α, IFN-γ, IL-6, and IL-10 than splenocytes from PBS-immunized mice. In addition, splenocytes from VirB8-immunized mice secreted IL-17(Th17) in response to VirB8 protein stimulation. Immunization with Omp19, VirB8, and HlyD did not induce significant increases in IL-4 secretion by stimulated splenocytes. These results showed that Omp19, VirB8, and HlyD can induce mice to produce TNF-α/IFN-γ, characterized by a Th1-type immune response. A Th17-type immune response induced by VirB8 was also detected.

After the mice were challenged with *B. abortus* S19, the protective immunity of the candidate antigens was evaluated by detecting the change in the organ bacterial load of mice. In the mouse model, the protection indexs for Omp19, VirB8 and HlyD antigen proteins combined with Freund's adjuvant in the spleen were 2.97, 2.36 and 1.09 and in the liver, the protection indexs were 1.29, 1.04 and 0.77, respectively (Table 2). The results of the challenge experiment showed that the Omp19, VirB8, and HlyD protective antigens could substantially reduce the load of *B. abortus* S19 in the organs of mice.

**Table 2:** **Protective efficacy conferred by antigen proteins against* B. abortus* S19 infection**

Vaccine Group (*n* = 6)	Adjuvant^a^	Log_10_ CFU (Spleen)^b^	Protection units	Log_10_ CFU (Liver)	Protection units
PBS	–	5.53 ± 0.19	0	5.7 ± 0.26	0
Omp19	FA/IFA/IFA	2.56 ± 0.21	2.97^***^	4.41 ± 0.22	1.29^***^
VirB8	FA/IFA/IFA	3.17 ± 0.49	2.36^***^	4.66 ± 0.46	1.04^**^
HlyD	FA/IFA/IFA	4.44 ± 0.15	1.09^**^	4.93 ± 0.15	0.77^*^
104 M	–	3.65 ± 0.35	1.88^***^	3.74 ± 0.16	1.96^***^

Protection units of immunized group is compared with that of PBS control with t-test, *p* < 0.05 is statistically significant. (*p < 0.05; **p < 0.01; ***p < 0.001)

^a^Adjuvant: FA, Freund’s adjuvant; IFA, incomplete Freund’s adjuvant.

^b^CFU, colony-forming units.

## Discussion

During the last two decades, various antigens have been evaluated from *Brucella* that have been shown to provide protection in mice. While these studies are highly promising, subunit vaccines using existing antigens cannot provide the same levels of protection as live attenuated vaccines [17]. Therefore, further investigation is needed to identify new antigens, in order to improve vaccine efficacy.

From the in silico predictions in this study, we screened 32 potential vaccine candidates including multiple outer membrane proteins and secretory system proteins. Membrane-related proteins often have important effects on the virulence and metabolism of bacteria, as these proteins are located on the bacterial surface and interact directly with immune system cells. Omp10, Omp16, and Omp19 are lipoproteins that possess various structures and functionality, ranging from bacterial physiology to pathogenesis mechanisms [55]. Omp25 is a virulence-related factor of *Brucella* that contributes to the intracellular survival of *Brucella* and chronic infection. The T4SS of *Brucella* is a well-known virulence factor that can directly regulate the secretion of effector proteins and plays a key role in the interaction between intracellular bacteria and the host [56]. VirB8 is thought to be an assembly factor that interacts with many other T4SS components in a mostly transient fashion [52]. VirB6 is a hydrophobic inner membrane protein containing five or more transmembrane helices that are believed to be located at or close to the translocation pore [52]. HlyD is a T1SS protein and specific component of the transport apparatus of alpha-haemolysin [53]. HlyD, HlyB, and TolC combine to form the three-component ABC transporter complex, creating a transmembrane channel or pore through which HlyA can be transferred directly to the extracellular medium. Other antigens found in our study included InpB, aromatic acid exporter family protein FusB, cell envelope biogenesis protein AsmA, heme transporter BhuA, and flagellin FliC. InpB is upregulated in response to environmental cues signalling vector-to-host transmission and is known to be a major virulence factor [57]. FusB can function as a metabolic relief valve, allowing elimination of certain compounds when these accumulate to high levels in the cell [58]. AsmA is involved in the assembly of outer membrane proteins that may play a role in lipopolysaccharide biogenesis [59]. BhuA is required by *B. abortus* for the maintenance of chronic infection in BALB/c mice [60]. Flagellin is a subunit protein that polymerises to form the flagellae, which are responsible for motility and chemotaxis. The FliC mutant in *Brucella* is attenuated in a murine model of infection, although *Brucella* spp. has been described as nonmotile bacteria [61].

By contrasting the similarity and dissimilarity of the candidate antigens identified in various studies, we observed similarities among the different RV strategies (Figure 4). For example, the outer membrane proteins Omp25 and BhuA on our list were also identified by Hisham, Vishnu, and He. One difference between previous studies and ours is that other researchers have applied decision-tree or filtering RV programs with traditional rules-based prediction. Proteins failing to comply with the set parameters were filtered at each step. An example is SCL, applied as the first major selection criterion in previous studies, limiting the target antigens to only surface-associated proteins. However, SCL might not be equally critical for *Brucella* in which cell-mediated immunity plays a major role. In our study, a compositive RV strategy based on several biological features was adopted for selecting novel potential vaccine candidates. Candidate proteins that did not satisfy the SLC criteria could still attain a reasonable score and were compensated by another set of selection criteria. For example, the plasma membrane protein InpB, predicted in our study as a candidate antigen, was not selected in previous studies as it did not meet their first major selection criterion, “SLC”. In contrast, candidate proteins that complied with the set parameters in previous studies but failed to attain a high composite score in our RV strategy were absent in our final list, such as LPS-assembly protein LptD.

In addition, compared with past studies, we focused on potential broad-spectrum antigens that can simultaneously target a variety of *Brucella* pathogens that have worldwide geographic distribution. Early studies of RV typically analysed few representative strains that are unfavourable targets for broad-spectrum therapeutics. Although Hisham and Ashhab combined pan-genome analysis with RV in their work, they did not consider the global geographic distribution of *Brucella* strains. Pathogenic species of *Brucella* are geographically distributed worldwide, including throughout Central Asia, Africa, South America, and the Mediterranean region [2]. Genomic resources from global analysis of a variety of pathogens geographically distributed worldwide can serve as a basis for identifying appropriate candidates for broad-spectrum vaccine antigens [62]. In our study, several non-broad-spectrum antigens identified in previous studies were absent from our antigen list, such as TonB-dependent receptor. Our results also provided a manageable list that includes some novel potential antigens that have not been previously reported. These findings can serve to extend the targets of *Brucella* vaccine candidates. Several limitations remain in the present compositive strategy using six biological features to predict protective antigens. Consideration of some other important factors including protein interaction and protein function, among others, may further increase the accuracy of protective antigen prediction.

Three antigens with representative scores and families, selected from among 32 candidate proteins, were verified and evaluated, to demonstrate the effectiveness of the present prediction method. Omp19, VirB8, and HlyD were observed to induce strong humoral and cellular immune responses. As *Brucella* spp. are facultative intracellular pathogens that can survive and replicate within macrophages, cell-mediated immunity is considered fundamental for protective immune response [63]. Our results showed that Omp19, VirB8, and HlyD can induce the body to produce TNF-α/IFN-γ, characterized by a Th1-type immune response. In particular, IFN-γ is essential for immune protection against *Brucella* infection that induce more polarization toward Th1 cells [64, 65]. In addition, functional TNF-α has been shown to link the proinflammatory response and adaptive immune response in *Brucella*-infected mice [66]. High levels of IL-6 were produced by splenocytes of Omp19-, HlyD-, and VirB8-immunized mice when re-exposed to the immunizing antigens. It has been showed that IL-6 promotes *B. abortus* clearance in macrophages and CD8 + T cell differentiation, priming the Th1 response during infection [67].

We have observed unmodified levels of specific IL-4 production among all group of mice, which indicated no involvement of this Th2 representative cytokine in the immune response against brucellosis [13, 68]. Significant levels of IL-10 in mice immunized with antigens were detected in this study. IL-10, a cytokine with broad immunoregulatory functions, was originally described as a unique product of Th2 cells but was later shown to be expressed in a variety of cell populations [69]. A Th17-type immune response induced by VirB8 was also observed that may contribute to the host defence against *Brucella* infection [70].

In summary, subcutaneous vaccination with Omp19, VirB8, and HlyD plus Freund’s adjuvant induced a strong humoral and Th1-oriented immune response. VirB8 could also induce a Th17 response. Moreover, the preliminary challenge experiments showed that Omp19, VirB8 and HlyD could substantially reduce the organ bacterial load of *B. abortus* S19 in mice and provide varying degrees of protection. These data provide encouragement that a recombinant protein-based vaccine can provide effective protection in a mouse model of brucellosis. Further validation of other predicted broad-spectrum antigens found in our study will follow.

A limitation in this study is the selection of hypovirulent strain *B. abortus* S19 as challenge strain for protection experiments [43, 71, 72]. *B. abortus* S19 was originally isolated as a virulent strain from a Jersey cow in 1923 and was found to become attenuated after being kept in the laboratory at room temperature for more than a year [41, 42]. It can also infect humans, causing typical features of brucellosis, including acute febrile illness. In addition, the challenge of pregnant mice using S19 reported an identical pathology, placentitis and septic fetal death, as with wild-type *B. abortus* infection [73, 74]. Preliminary protection experiments using S19 instead of wild-type *B. abortus* are reduced cost and safety, since BSL-3 small animal containment facilities are not required according to biosafety procedures in China [75]. Additional studies are being undertaken to evaluation the protective efficacy of antigens against infection with different pathogenic *Brucella* species (*B. abortus*, *B. melitensis* and *B. suis*).

With the aim of finding broad-spectrum protein candidates of pathogenic *Brucella* spp. with worldwide geographic distribution for vaccine development, we adapted and optimized a compositive RV methodology. Protein candidates from the core proteome of pathogenic *Brucella* spp. were screened and scored according to six biological features that are strongly associated with protective antigenicity. The 32 top-ranked potential vaccine candidates were screened via compositive RV analyses. The outer membrane protein Omp19 (used as a positive control), T4SS protein VirB8, and T1SS protein HlyD were selected for preliminary verification. In a mouse model, Omp19, VirB8 and HlyD in the presence of Freund’s adjuvant could significantly reduce the *B. abortus* S19 colonization in spleen and liver tissues. Further evaluation is needed to identify the levels of protection conferred by the vaccine antigens against wild-type pathogenic *Brucella* species challenge. Compared with previous reports, our findings provide a manageable list of potential broad-spectrum antigens for developing a potent vaccine against brucellosis. We also demonstrated the effectiveness of this unique strategy in this work. The simplified approach toward vaccine candidate identification used in this study is widely applicable to other pathogens.

## Supplementary Information

**Additional file 1: List of 213 pathogenic** ***Brucella* spp. strains with clear genetic isolation information**.

**Additional file 2: The composite score for all non-host-homology** ***Brucella* proteins using compositive reverse vaccinology methodology**.

## References

[1] Pappas G, Akritidis N, Bosilkovski M, Tsianos E (2005). Brucellosis. N Engl J Med.

[2] Moreno E (2014). Retrospective and prospective perspectives on zoonotic brucellosis. Front Microbiol.

[3] Byndloss MX, Tsolis RM (2016). *Brucella* spp. virulence factors and immunity. Annu Rev Anim Biosci.

[4] Pappas G, Papadimitriou P, Akritidis N, Christou L, Tsianos EV (2006). The new global map of human brucellosis. Lancet Infect Dis.

[5] Lalsiamthara J, Lee JH (2017). Development and trial of vaccines against *Brucella*. J Vet Sci.

[6] Dorneles EM, Sriranganathan N, Lage AP (2015). Recent advances in *Brucella abortus* vaccines. Vet Res.

[7] Pasquevich KA, Estein SM, Garcia Samartino C, Zwerdling A, Coria LM, Barrionuevo P, Fossati CA, Giambartolomei GH, Cassataro J (2009). Immunization with recombinant *Brucella* species outer membrane protein Omp16 or Omp19 in adjuvant induces specific CD4+ and CD8+ T cells as well as systemic and oral protection against *Brucella abortus* infection. Infect Immun.

[8] Goel D, Rajendran V, Ghosh PC, Bhatnagar R (2013). Cell mediated immune response after challenge in Omp25 liposome immunized mice contributes to protection against virulent *Brucella abortus* 544. Vaccine.

[9] Mallick AI, Singha H, Chaudhuri P, Nadeem A, Khan SA, Dar KA, Owais M (2007). Liposomised recombinant ribosomal L7/L12 protein protects BALB/c mice against *Brucella abortus* 544 infection. Vaccine.

[10] Gupta S, Mohan S, Somani VK, Aggarwal S, Bhatnagar R (2020). Simultaneous immunization with Omp25 and L7/L12 provides protection against Brucellosis in mice. Pathogens.

[11] Al-Mariri A, Mahmoud NH, Hammoud R (2012). Efficacy evaluation of live *Escherichia coli* expression *Brucella* P39 protein combined with CpG oligodeoxynucleotides vaccine against *Brucella melitensis* 16M, in BALB/c mice. Biologicals.

[12] Hop HT, Arayan LT, Huy TXN, Reyes AWB, Min W, Lee HJ, Park SJ, Chang HH, Kim S (2018). Immunization of BALB/c mice with a combination of four recombinant *Brucella abortus* proteins, AspC, Dps, InpB and Ndk, confers a marked protection against a virulent strain of *Brucella abortus*. Vaccine.

[13] Ghasemi A, Jeddi-Tehrani M, Mautner J, Salari MH, Zarnani AH (2015). Simultaneous immunization of mice with Omp31 and TF provides protection against *Brucella melitensis* infection. Vaccine.

[14] Wang X, An C, Yang M, Li X, Ke Y, Lei S, Xu X, Yu J, Ren H, Du X, Wang Z, Qiu Y, Liu B, Chen Z (2015). Immunization with individual proteins of the Lrp/AsnC family induces protection against *Brucella melitensis* 16M challenges in mice. Front Microbiol.

[15] Hou H, Liu X, Peng Q (2019). The advances in brucellosis vaccines. Vaccine.

[16] Masjedian Jezi F, Razavi S, Mirnejad R, Zamani K (2019). Immunogenic and protective antigens of *Brucella* as vaccine candidates. Comp Immunol Microbiol Infect Dis.

[17] Carvalho TF, Haddad JP, Paixao TA, Santos RL (2016). Meta-analysis and advancement of brucellosis vaccinology. PLoS One.

[18] Delany I, Rappuoli R, Seib KL (2013). Vaccines, reverse vaccinology, and bacterial pathogenesis. Cold Spring Harb Perspect Med.

[19] Moxon R, Reche PA, Rappuoli R (2019). Editorial: reverse vaccinology. Front Immunol.

[20] He Y, Xiang Z (2010). Bioinformatics analysis of *Brucella* vaccines and vaccine targets using VIOLIN. Immunome Res.

[21] Gomez G, Pei J, Mwangi W, Adams LG, Rice-Ficht A, Ficht TA (2013). Immunogenic and invasive properties of *Brucella melitensis* 16M outer membrane protein vaccine candidates identified via a reverse vaccinology approach. PLoS One.

[22] Vishnu US, Sankarasubramanian J, Gunasekaran P, Rajendhran J (2015). Novel vaccine candidates against *Brucella melitensis* identified through reverse vaccinology approach. OMICS.

[23] Hisham Y, Ashhab Y (2018). Identification of cross-protective potential antigens against pathogenic Brucella spp. through combining pan-genome analysis with reverse vaccinology. J Immunol Res.

[24] Ong E, Wong MU, He Y (2017). Identification of new features from known bacterial protective vaccine antigens enhances rational vaccine design. Front Immunol.

[25] Dalsass M, Brozzi A, Medini D, Rappuoli R (2019). Comparison of open-source reverse vaccinology programs for bacterial vaccine antigen discovery. Front Immunol.

[26] National Center for Biotechnology Information. https://www.ncbi.nlm.nih.gov

[27] Chaudhari NM, Gupta VK, Dutta C (2016). BPGA- an ultra-fast pan-genome analysis pipeline. Sci Rep.

[28] Camacho C, Coulouris G, Avagyan V, Ma N, Papadopoulos J, Bealer K, Madden TL (2009). BLAST+: architecture and applications. BMC Bioinformatics.

[29] Yu CS, Cheng CW, Su WC, Chang KC, Huang SW, Hwang JK, Lu CH (2014). CELLO2GO: a web server for protein subCELlular LOcalization prediction with functional gene ontology annotation. PLoS One.

[30] Yang B, Sayers S, Xiang Z, He Y (2011). Protegen: a web-based protective antigen database and analysis system. Nucleic Acids Res.

[31] He Y, Racz R, Sayers S, Lin Y, Todd T, Hur J, Li X, Patel M, Zhao B, Chung M, Ostrow J, Sylora A, Dungarani P, Ulysse G, Kochhar K, Vidri B, Strait K, Jourdian GW, Xiang Z (2014). Updates on the web-based VIOLIN vaccine database and analysis system. Nucleic Acids Res.

[32] Doytchinova IA, Flower DR (2007). VaxiJen: a server for prediction of protective antigens, tumour antigens and subunit vaccines. BMC Bioinform.

[33] Santos AR, Pereira VB, Barbosa E, Baumbach J, Pauling J, Rottger R, Turk MZ, Silva A, Miyoshi A, Azevedo V (2013). Mature Epitope Density–a strategy for target selection based on immunoinformatics and exported prokaryotic proteins. BMC Genomics.

[34] Gupta A, Kapil R, Dhakan DB, Sharma VK (2014). MP3: a software tool for the prediction of pathogenic proteins in genomic and metagenomic data. PLoS One.

[35] He Y, Xiang Z, Mobley HL (2010). Vaxign: the first web-based vaccine design program for reverse vaccinology and applications for vaccine development. J Biomed Biotechnol.

[36] Huerta-Cepas J, Forslund K, Coelho LP, Szklarczyk D, Jensen LJ, von Mering C, Bork P (2017). Fast genome-wide functional annotation through orthology assignment by eggNOG-mapper. Mol Biol Evol.

[37] Kelley LA, Mezulis S, Yates CM, Wass MN, Sternberg MJ (2015). The Phyre2 web portal for protein modeling, prediction and analysis. Nat Protoc.

[38] Darzentas N (2010). Circoletto: visualizing sequence similarity with Circos. Bioinformatics.

[39] Yu D, Hui Y, Zai X, Xu J, Liang L, Wang B, Yue J, Li S (2015). Comparative genomic analysis of *Brucella abortus* vaccine strain 104M reveals a set of candidate genes associated with its virulence attenuation. Virulence.

[40] Wang Y, Ke Y, Wang Z, Yuan X, Qiu Y, Zhen Q, Xu J, Li T, Wang D, Huang L, Chen Z (2012). Genome sequences of three live attenuated vaccine strains of *Brucella* species and implications for pathogenesis and differential diagnosis. J Bacteriol.

[41] Thomas EL, Bracewell CD, Corbel MJ (1981). Characterisation of *Brucella abortus* strain 19 cultures. Vet Rec.

[42] Qian M, Zhao T, Li R, Yang Q, Yu R, Yin Y, Zai X, Li Y, Zhang J, Xu J, Chen W (2018). Targeting the R domain of coagulase by active vaccination protects mice against lethal *Staphylococcus aureus* infection. Microbes Infect.

[43] Lowry JE, Isaak DD, Leonhardt JA, Vernati G, Pate JC, Andrews GP (2011). Vaccination with *Brucella abortus* recombinant in vivo-induced antigens reduces bacterial load and promotes clearance in a mouse model for infection. PLoS One.

[44] Flower DR, Macdonald IK, Ramakrishnan K, Davies MN, Doytchinova IA (2010). Computer aided selection of candidate vaccine antigens. Immunome Res.

[45] Kline KA, Falker S, Dahlberg S, Normark S, Henriques-Normark B (2009). Bacterial adhesins in host-microbe interactions. Cell Host Microbe.

[46] Castaneda-Roldan EI, Avelino-Flores F, Dall'Agnol M, Freer E, Cedillo L, Dornand J, Giron JA (2004). Adherence of *Brucella* to human epithelial cells and macrophages is mediated by sialic acid residues. Cell Microbiol.

[47] Munoz Gonzalez F, Sycz G, Alonso Paiva IM, Linke D, Zorreguieta A, Baldi PC, Ferrero MC (2019). The BtaF adhesin is necessary for full virulence during respiratory infection by *Brucella suis* and is a novel immunogen for nasal vaccination against *Brucella* infection. Front Immunol.

[48] Tibor A, Wansard V, Bielartz V, Delrue RM, Danese I, Michel P, Walravens K, Godfroid J, Letesson JJ (2002). Effect of omp10 or omp19 deletion on *Brucella abortus* outer membrane properties and virulence in mice. Infect Immun.

[49] Risso GS, Carabajal MV, Bruno LA, Ibanez AE, Coria LM, Pasquevich KA, Lee SJ, McSorley SJ, Briones G, Cassataro J (2017). U-Omp19 from *Brucella abortus* is a useful adjuvant for vaccine formulations against *Salmonella* infection in mice. Front Immunol.

[50] Pasquevich KA, Carabajal MV, Guaimas FF, Bruno L, Roset MS, Coria LM, Rey Serrantes DA, Comerci DJ, Cassataro J (2019). Omp19 enables *Brucella abortus* to evade the antimicrobial activity from host’s proteolytic defense system. Front Immunol.

[51] Sharifahmadian M, Nlend IU, Lecoq L, Omichinski JG, Baron C (2017). The type IV secretion system core component VirB8 interacts via the beta1-strand with VirB10. FEBS Lett.

[52] Casu B, Mary C, Sverzhinsky A, Fouillen A, Nanci A, Baron C (2018). VirB8 homolog TraE from plasmid pKM101 forms a hexameric ring structure and interacts with the VirB6 homolog TraD. Proc Natl Acad Sci U S A.

[53] Bielaszewska M, Aldick T, Bauwens A, Karch H (2014). Hemolysin of enterohemorrhagic *Escherichia coli*: structure, transport, biological activity and putative role in virulence. Int J Med Microbiol.

[54] Dorneles EMS, Teixeira-Carvalho A, Araújo MSS, Sriranganathan N, Lage AP (2015). Immune response triggered by *Brucella abortus* following infection or vaccination. Vaccine.

[55] Goolab S, Roth RL, van Heerden H, Crampton MC (2015). Analyzing the molecular mechanism of lipoprotein localization in *Brucella*. Front Microbiol.

[56] Lacerda TLS, Salcedo SP, Gorvel J-P (2013). *Brucella* T4SS: the VIP pass inside host cells. Curr Opin Microbiol.

[57] Coleman SA, Minnick MF (2003). Differential expression of the invasion-associated locus B (*ialB*) gene of *Bartonella bacilliformis* in response to environmental cues. Microb Pathog.

[58] Van Dyk TK, Templeton LJ, Cantera KA, Sharpe PL, Sariaslani FS (2004). Characterization of the *Escherichia coli* AaeAB efflux pump: a metabolic relief valve. J Bacteriol.

[59] Deng M, Misra R (1996). Examination of AsmA and its effect on the assembly of *Escherichia coli* outer membrane proteins. Mol Microbiol.

[60] Paulley JT, Anderson ES, Roop RM (2007). *Brucella abortus* requires the heme transporter BhuA for maintenance of chronic infection in BALB/c mice. Infect Immun.

[61] Fretin D, Fauconnier A, Kohler S, Halling S, Leonard S, Nijskens C, Ferooz J, Lestrate P, Delrue RM, Danese I, Vandenhaute J, Tibor A, DeBolle X, Letesson JJ (2005). The sheathed flagellum of *Brucella melitensis* is involved in persistence in a murine model of infection. Cell Microbiol.

[62] Davies MR, McIntyre L, Mutreja A, Lacey JA, Lees JA, Towers RJ, Duchene S, Smeesters PR, Frost HR, Price DJ, Holden MTG, David S, Giffard PM, Worthing KA, Seale AC, Berkley JA, Harris SR, Rivera-Hernandez T, Berking O, Cork AJ, Torres R, Lithgow T, Strugnell RA, Bergmann R, Nitsche-Schmitz P, Chhatwal GS, Bentley SD, Fraser JD, Moreland NJ, Carapetis JR (2019). Atlas of group A streptococcal vaccine candidates compiled using large-scale comparative genomics. Nat Genet.

[63] Martirosyan A, Moreno E, Gorvel JP (2011). An evolutionary strategy for a stealthy intracellular *Brucella* pathogen. Immunol Rev.

[64] Murphy EA, Sathiyaseelan J, Parent MA, Zou B, Baldwin CL (2001). Interferon-gamma is crucial for surviving a *Brucella abortus* infection in both resistant C57BL/6 and susceptible BALB/c mice. Immunology.

[65] Vitry MA, De Trez C, Goriely S, Dumoutier L, Akira S, Ryffel B, Carlier Y, Letesson JJ, Muraille E (2012). Crucial role of gamma interferon-producing CD4+ Th1 cells but dispensable function of CD8+ T cell, B cell, Th2, and Th17 responses in the control of *Brucella melitensis* infection in mice. Infect Immun.

[66] Zhan Y, Cheers C (1998). Control of IL-12 and IFN-gamma production in response to live or dead bacteria by TNF and other factors. J Immunol.

[67] Hop HT, Huy TXN, Reyes AWB, Arayan LT, Vu SH, Min W, Lee HJ, Kang CK, Kim DH, Tark DS, Kim S (2019). Interleukin 6 promotes *Brucella abortus* clearance by controlling bactericidal activity of macrophages and CD8(+) T cell differentiation. Infect Immun.

[68] Paul S, Peddayelachagiri BV, Nagaraj S, Konduru B, Batra HV (2018). Protective and therapeutic efficacy study of divalent fusion protein rL7/L12-Omp25 against *B. abortus* 544 in presence of IFNγ. Appl Microbiol Biotechnol.

[69] Jankovic D, Kugler DG, Sher A (2010). IL-10 production by CD4+ effector T cells: a mechanism for self-regulation. Mucosal Immunol.

[70] Clapp B, Skyberg JA, Yang X, Thornburg T, Walters N, Pascual DW (2011). Protective live oral brucellosis vaccines stimulate Th1 and th17 cell responses. Infect Immun.

[71] Chen B, Liu B, Zhao Z, Wang G (2019). Evaluation of a DNA vaccine encoding *Brucella* BvrR in BALB/c mice. Mol Med Rep.

[72] Huang J, Pan C, Sun P, Feng E, Wu J, Zhu L, Wang HL (2020). Application of an *O*-linked glycosylation system in *Yersinia enterocolitica* serotype O:9 to generate a new candidate vaccine against *Brucella abortus*. Microorganisms.

[73] Tobias L, Schurig GG, Cordes DO (1992). Comparative behaviour of *Brucella abortus* strains 19 and RB51 in the pregnant mouse. Res Vet Sci.

[74] Tobias L, Cordes DO, Schurig GG (1993). Placental pathology of the pregnant mouse inoculated with *Brucella abortus* strain 2308. Vet Pathol.

[75] The Ministry of Health in China (2006) List of pathogens contagious to humans. http://www.nhc.gov.cn/qjjys/s3589/200804/f0840e5958ea40c68ba0cfda9cc138ac.shtml. Accessed 27 Jan 2006

